# Box–Behnken design for removal of uranium(VI) from aqueous solution using poly(ethylene glycol) based dicationic ionic liquid impregnated chitosan

**DOI:** 10.3906/kim-1911-73

**Published:** 2020-06-01

**Authors:** Süleyman İNAN, Taşkın MUMCU, Serap SEYHAN BOZKURT

**Affiliations:** 1 Institute of Nuclear Sciences, Ege University, İzmir Turkey; 2 Department of Chemistry, Faculty of Science, Dokuz Eylül University, İzmir Turkey

**Keywords:** Uranium(VI), sorption, dicationic ionic liquid, poly(ethylene glycol), chitosan, Box–Behnken design

## Abstract

Poly(ethylene glycol) bis(methylimidazolium) di[bis(trifluoromethylsulfonyl)imide] was synthesized as an ionic liquid and impregnated onto chitosan. The removal of uranium(VI) ions from aqueous solution was investigated with batch sorption tests using ionic liquid impregnated chitosan. Response surface methodology based on 3 level Box–Behnken design was applied to analyze the effect of initial pH (4–6), initial concentration (20–60 mg L^-1^), contact time (15–105 min), and temperature (30–50 °C) on the uptake capacity of uranium(VI). Main effect of initial concentration, quadratic effect of contact time, and dual effect of initial pH and contact time were found statistically significant based on analysis of variance (ANOVA). Probability F-value (F = 1.49 ×10^-6^) and correlation coefficient (R2 = 0.96) point out that the proposed model is compatible with experimental data. The maximum uptake capacity of uranium(VI) was found as 28.48 mg g^-1^ at initial pH 4, initial concentration 60 mg L^-1^, contact time of 70 min, and a temperature of 50 °C. Sorption kinetics followed a pseudo-second-order model and Freundlich model was obtained to fit the sorption data. The presence of competing ions slightly reduced uranium(VI) sorption and the selectivity order can be given as UO_2_^2+^>Zn^2+^>Ni^2+^.

## 1. Introduction

Uranium is a naturally occurring radioactive element and chemically toxic heavy metal. During the stages of nuclear fuel production such as mining, purification, and enrichment, large amount of uranium polluted wastewater is produced. Additional uranium sources are natural deposits and depleted uranium ammunitions [1]. Uranium exists mostly in the valence states of uranium(IV) and uranium(VI) depending on the environment. However, the oxidized state uranium(VI) can highly migrate and it is more soluble. Therefore, the direct discharge of uranium polluted waste streams is hazardous for environment and human health. Uranium can enter the human body via food chain. It can cause harmful effects on skin, kidneys, liver, and may even lead to death [2]. Consequently, it is highly important to remove uranium from wastewater prior to discharge and to prevent its mobilization into the environment.

Several methods including ion exchange, adsorption, chemical precipitation and solvent extraction [3–6] have been used for the separation of uranium from aqueous waste streams. These methods have some disadvantages due to technical, economic, and environmental issues. Among those methods, solvent extraction is one of the most commonly used methods for the separation of uranium ions. It has recently attracted more attention with the use of ionic liquids (ILs). ILs have well-known properties such as low volatility and vapor pressure, high thermal stability, ionic conductivity, and miscibility with solvents [7,8]. However, liquid–liquid extraction has also some disadvantages such as using large quantities of ILs and the loss of IL in the aqueous phase because of insufficient phase separations [9]. The utilization of IL in solid–liquid separation processes can be proposed as a solution to overcome the problems of the liquid–liquid extractions. By the immobilization and impregnation of ILs in a suitable solid support, the consumed amount of ILs can be minimized and the loss of ILs in the aqueous phase can be reduced. Moreover, the solid support can enhance the properties of ILs for the metal ion removal [10–12].

Many types of organic and inorganic support materials have been under research for the impregnation of ILs. Macroporous organic polymers such as Amberlite type resins [13–15] (XAD 2, XAD 4, XAD 7, XAD 8) are well-known with their high surface area, uniform pore size distribution, and good mechanical and chemical stability. However, their thermal degradation generates toxic compounds. This issue could be fixed by using biopolymers such as alginate [16], cellulose [17,18], and chitosan [9,19] as a solid support. These polymers have a thermal degradation which is less harmful to the environment in comparison with synthetic resins.

Chitosan, which is a natural biopolymer, is recognized as an excellent sorbent for heavy metal ions because of its high surface area and active sites such as amino and hydroxyl groups [10,20]. It is utilized extensively for the sorption of metal ions in aqueous solutions [21,22]. The properties of chitosan can be modified and improved, so it can be used in a large number of applications. Wang et al. [23] investigated uranium(VI) adsorption behavior on cross-linked chitosan. Liu et al. [24] prepared chitosan/ZIF-8 composite beads for the efficient removal of U(VI). Lupa et al. [9] conducted a research on the adsorption of cesium and strontium ions by 1-ethyl-3-methyl imidazolium chloride impregnated chitosan. Eliodorio et al. [25] investigated the chromium adsorption behaviors of chitosan treated with two new ILs. Sorption kinetics of zinc and nickel on modified chitosan were investigated by Tripathi et al. [26].

In a surface response experiment, the independent variables or factors can be varied over a continuous range. The aim is to determine the factor settings that produce a maximum or minimum response or to map the relationship between the response and factors. Response surface methods have found considerable use in industry especially in chemical processes where the reaction yield or cost of production can be optimized as a function of controllable process factors. Box and Behnken (1960) developed some three level designs that will allow estimation of the general quadratic model. These designs consist of 22 factorials in each pair of factors with all other factors held constant at their mid-level plus a few center points. Box Behnken designs require factors be varied over three levels and they usually require less total runs than the central composite design. This may make experimentation less costly [27].

In the present study, a novel IL, poly(ethylene glycol) bis(methylimidazolium) di[bis(trifluoromethylsulfon yl)imide] was synthesized and impregnated onto chitosan. The prepared sorbent was characterized by FTIR, SEM-EDX and TGA analysis. Box–Behnken design (BBD) was employed to analyze the effect of initial pH, initial concentration, contact time and temperature on the uptake capacity of uranium(VI). Sorption kinetics, isotherms and the effect of competing ions in mixed metal solutions were studied. Desorption experiments were carried out.

## 2. Materials and methods

### 2.1. Reagents and instrumentation

Uranyl nitrate hexahydrate (UO2 (NO3)2 .6H2 O) and bis(trifluoromethane)sulfonimide lithium salt (CF3 SO2 NLiSO2 CF3) were purchased from Sigma-Aldrich (Switzerland). 1-methylimidazolium (C4 H7 N2) was supplied by Merck (Germany). Chitosan was supplied by Sigma-Aldrich (Japan). Poly(ethylene glycol) 600 (H(OCH2 CH2)n OH) was obtained from Alfa Aesar (Germany). Pyridine (C5 H5 N) was purchased from Carlo Erba (France). Thionyl chloride (SOCl2) was obtained from Acros Organics (Germany). All solvents used were of analytical grade and were supplied by Merck (Germany). The desired concentration of test solutions were obtained by diluting stock solution in appropriate volumes. pH of the test solutions were adjusted by adding nitric acid and ammonia solutions.

Fourier transform infrared (FTIR) data (400-4000 cm−1) were acquired by Spectrum Two model FTIR spectrometer with attenuated total reflectance accessory (Perkin Elmer, USA). The thermal stability and weight loss of IL impregnated chitosan were investigated in the temperature range 20-800 ºC using SDT Q600 model thermogravimetric analyzer (TGA) & differential scanning calorimeter (DSC), (TA Instruments, USA). The analysis was conducted with a linear heating rate of 10 ºC min−1 in a nitrogen gas flow. Scanning electron microscopy (SEM)-energy dispersive X-ray analysis (EDX) was performed using Apreo S model SEM coupled with EDX detector (Thermo Scientific, USA). The analysis of uranium(VI) was carried out by Optima 2000DV model Inductively Coupled Plasma Optical Emission Spectrometer (ICP-OES), (Perkin Elmer, USA).

### 2.2. Preparation of IL impregnated chitosan

As a first step, poly(ethylene glycol) was converted to poly(ethylene glycol) dichloride. Consequently, this material was reacted with 1-methylimidazolium and poly(ethylene glycol) bis(methyimidazolium) dichloride IL was obtained. This IL was mixed with bis(trifluoromethane)sulfonimide lithium salt and poly(ethylene glycol) bis(methylimidazolium) di[bis(trifluoromethylsulfonyl)imide] was synthesized (Figure 1). Then the synthesized IL was impregnated onto chitosan.

**Figure:**
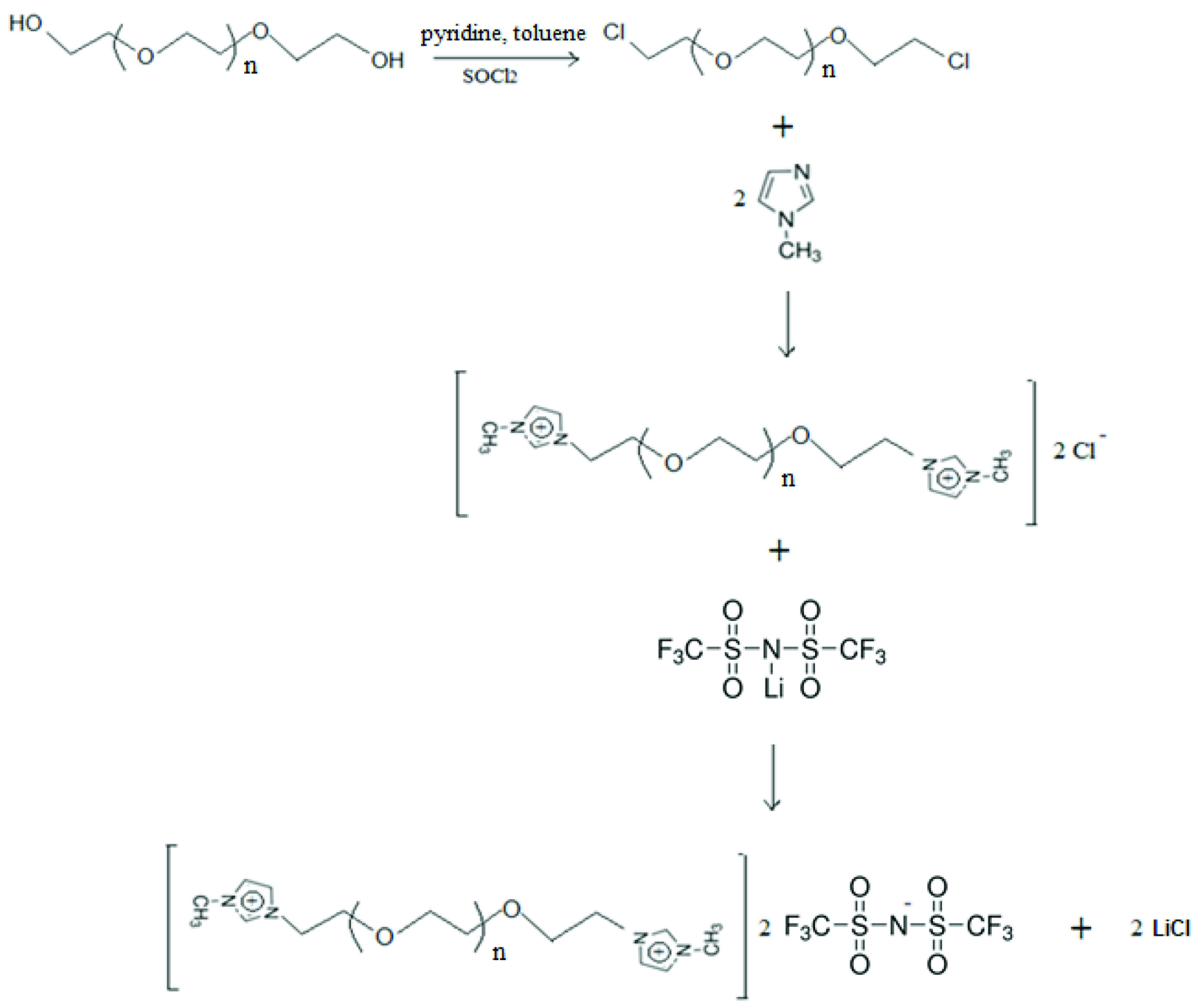
The synthesis of poly(ethylene glycol) bis(methylimidazolium) di[bis(trifluoromethyl)sulfonyl)imide].

#### 2.2.1. Preparation of poly(ethylene glycol) dichloride

Poly(ethylene glycol) 600 and pyridine were dissolved in toluene and the resulting mixture was heated up to 87 ºC and thionyl chloride was slowly added. The obtained mixture was stirred at 87 ºC for 15 hours. The resulting solid was filtered and the solvent was removed from the mixture by evaporator [28]. The poly(ethylene glycol) dichloride was obtained in 90% yield.

#### 2.2.2. Preparation of poly(ethylene glycol) based dicationic ionic liquid

The synthesized poly(ethylene glycol) dichloride and 1-methylimidazolium was stirred at 80 ºC for 16 hours at a molar ratio of 1:2. Excess of 1-methylimidazolium was then removed by washing with ethyl acetate. The obtained poly(ethylene glycol) bis(methyimidazolium) dichloride was washed with ethyl ether and purified deionized water and dried in a vacuum oven at ~60-65 ºC [28]. Then this IL was mixed with bis(trifluoromethane)sulfo nimide lithium salt in methanol and stirred for 24 hours at room temperature. The product was separated and washed with deionized water. After that, poly(ethylene glycol) bis(methylimidazolium) di[bis(trifluoromethylsulf onyl)imide] was dried in a vacuum oven at 60 ºC [29] and 65% efficiency was achieved from the reaction.

#### 2.2.3. Preparation of sorbent

1 g of chitosan and 45 mL of ultra pure water were mixed and pH of the solution was adjusted to 3 with 1 mol L−1 HCl. The mixture was stirred for 1 hour at room temperature. On the other hand, 0.75 gpoly(ethylene glycol) bis(methylimidazolium) di[bis(trifluoromethylsulfonyl)imide] was dissolved in 5 mL of dichloromethane. Following this step, IL solution was added to chitosan solution and the mixture was stirred for 24 hours at room temperature. After that, the mixture was filtered and the sorbent was washed with dichloromethane and dried in a vacuum oven at ~50-60 ºC [30]. Thus, the sorbent to be used for the sorption of uranium(VI) ions was prepared.

### 2.3. Design of experiments

Box and Behnken (1960) have offered some three level designs by associating 2k factorials with incomplete block designs [31]. In this study, BBD has been used to optimize the sorption conditions. The effect of four independent process variables (initial pH (X1) , initial concentration (X2) , contact time (X3) , and temperature (X4)) on uranium(VI) sorption was investigated by making 27 experiments in a temperature controlled shaker at 150 rpm. Batch sorption studies of uranium(VI) ions on IL impregnated chitosan were carried out with 50 mg sorbent in 25 mL of liquid phase. The samples were then filtered with qualitative filter paper and uranium(VI) concentrations in the solution were analyzed before and after equilibrium by ICP-OES. The operating conditions of ICP-OES are summarized in Table 1. The sorption capacity (Q) was calculated using Eq. (1):

(1)Capacity(Q)=(C0-Ce)xVm

**Table 1:** Operating conditions of ICP-OES.

Parameter	Condition
Plasma gas flow rate (L min^-1^)	15
Auxiliary gas flow rate (L min^-1^)	0.2
Nebulizer gas flow rate (L min^-1^)	80
RF power (W)	1000
Sample flow rate (mL min^-1^)	1.5
Read delay (s)	15
Replicates	2
Element U Zn Ni	Detection wavelength (nm) 385.958 206.200 231.604

where *C*_0_ and *C_e_* are the initial and equilibrium concentrations of uranium(VI) ion in solution (mg L^-1^). *V* is the volume (mL) and m is the mass of the sorbent (g).

The range and levels of variables (low, center, high) examined in this study are presented in Table 2.

**Table 2:** 

*Variable*		-1	0	+1
Initial pH	X_1_	4	5	6
Initial concentration (mg L^-1^)	X_2_	20	40	60
Contact time (min)	X_3_	15	60	105
Temperature (°C)	X_4_	30	40	50

The design matrix of four variables was changed at three levels (–1, 0, +1). The model equation including linear and quadratic terms for the estimation of optimum response is shown in Eq. (2).

(2)yi=b0+bi+biiXii2+bijXiXj

where *y_i_* represents the response (uranium(VI) sorption capacity), *X_i_* and *X_j_* independent coded variables, and *b_0_* , *b_i_* , *b_ii_* , *b_ij_* the intercept term, linear, quadratic, and dual interaction effects, respectively. Design-Expert 12 software was used to perform statistical, regression, and graphical analysis of the data.

The second-order polynomial equation can be written as in Eq. (3).

(3)y=b0+b1X1+b2X2+b3X3+b4X4+b11X12+b22X22+b33X32+b44X42+b12X1X2+b12X1X2+b13X1X3+b23X2X3+b24X2X4+b34X3X4

## 3. Results and discussion

### 3.1. Characterization studies

The FTIR spectrum of poly(ethylene glycol) bis(methylimidazolium) di[bis(trifluoromethylsulfonyl)imide] was compared with the spectrum of starting materials. The characteristic peaks in the FTIR spectrum of dicationic IL were observed as follows FTIR (cm^-1^): ν_=C-H_ = 3125, ν_C-H_ = 2928-2875, ν_C=N_ = 1562, ν_C=C_ = 1474, ν_C-F_ = 1428, ν_C-O_ = 1195, ν_C-N_ = 1142, ν_C-C_ = 1057, ν_S=O_ = 1035, ν_C-S_ =735.

The FTIR spectrum of IL impregnated chitosan was also compared with the spectrum of IL. The characteristic peaks were as follows FTIR (cm^-1^): ν_OH_ and NH_2_ = 3335, ν_=C-H_ = 3299, ν_C-H_ = 2875, ν_C=N_ = 1654, ν_C=C_ = 1579, ν_C-F_ = 1349, ν_C-O_ = 1196, ν_C-N_ = 1148, ν_C-C_ = 1050, ν_S=O_ = 1026, ν_C-S_ = 892. The presence of vibration bands of =C-H, C=N, C=C, C-F, C-O, S=O, and C-S groups in the FTIR spectrum of IL-based chitosan sorbent showed that the IL was impregnated onto chitosan. Moreover, the vibration bands of C-F, S=O, and C-S groups in the sorbent changed according to the IL.

**Figure 2:**
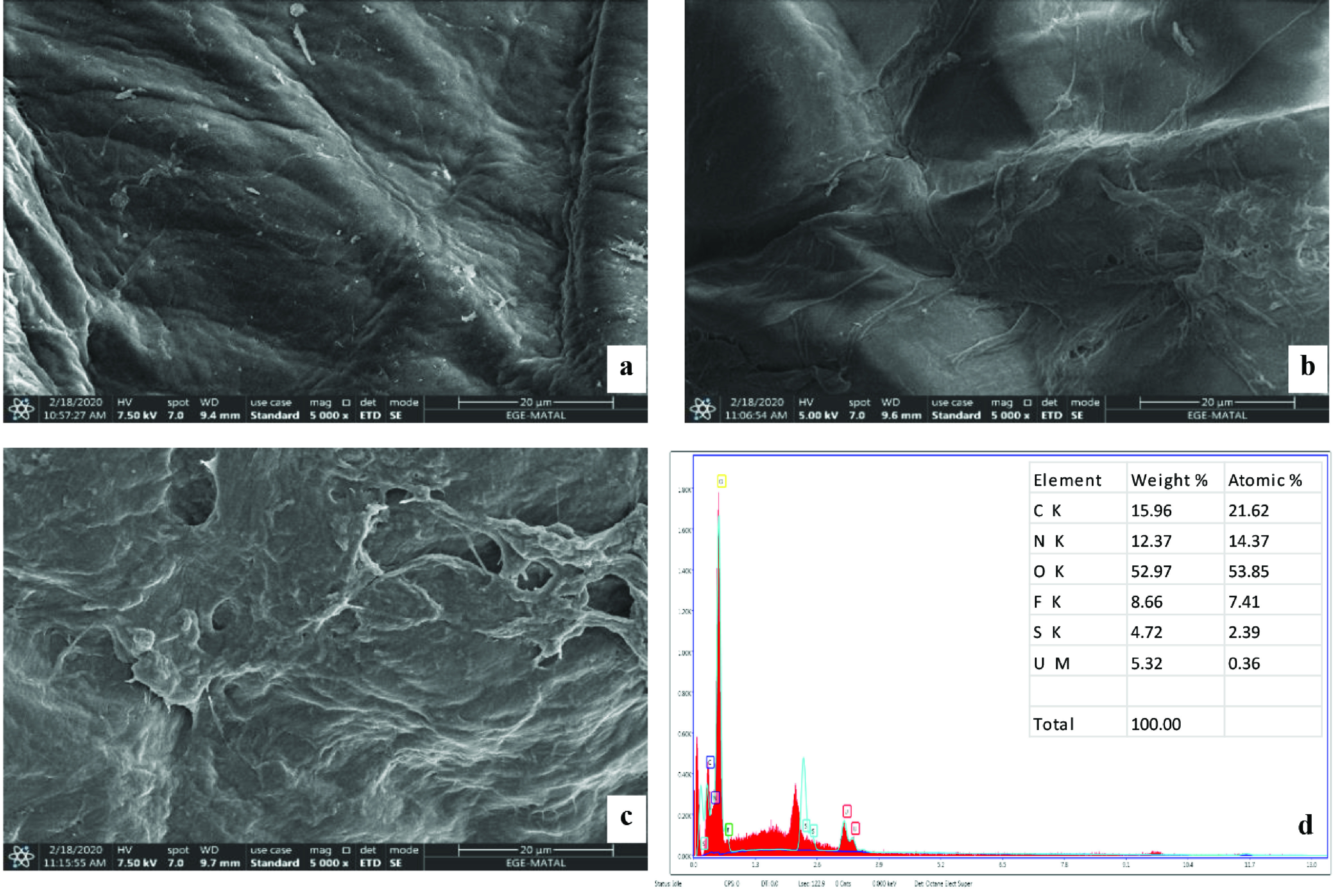
SEM images with 5000×magnification; chitosan (a), IL-impregnated chitosan (b), IL impregnated chitosan after uranium(VI) sorption (c), and EDX spectra of IL-impregnated chitosan after uranium(VI) sorption (d).

The surface characteristics of chitosan, IL-impregnated chitosan, and IL-impregnated chitosan after uranium(VI) sorption were examined with SEM analysis. While the morphology of chitosan has a layered structure (Figure 2a), the surface has become porous after the impregnation of IL (Figure 2b). Changes on the surface of the sorbent after sorption showed that uranium(VI) ions were successfully sorbed (Figure 2c). Additionally, the presence of uranium(VI) on the surface of IL impregnated chitosan was confirmed by energy dispersive X-ray spectroscopy (EDX) measurement. The determination of carbon, oxygen, nitrogen, fluorine, sulfur, and uranium proved that IL was impregnated onto chitosan and uranium(VI) ions are sorbed on the sorbent (Figure 2d).

The thermal stability of IL-impregnated chitosan was also investigated with TGA curve. It was seen that the prepared sorbent was stable at the studied temperature.

In order to examine the stability of IL-impregnated chitosan in acidic solutions, weighed amount of particles were immersed into the nitric acid solutions in the pH range of 3–5 for 5 days. The particles were dried at 40 °C and the weight losses (%) were calculated. It was determined that the IL impregnated chitosan has a weight loss of 1.6%, 2.2%, and 2.0% at pH 3, pH 4, and pH 5, respectively. The results prove that ILimpregnated chitosan has a good chemical stability under experimental conditions. However, chemical properties of the sorbent should be improved for the utilization in concentrated acidic solutions.

### 3.2. Box–Behnken design

BBD consists of 24 factorial and 3 replicate points. Experimental variables in coded and actual form along with predicted and actual responses were shown in Table 3. The second-order polynomial equation indicated the relation between independent variables and response. The regression coefficients were determined using a second-order polynomial equation (Eq. 4).

(4)y=18.96-0.31X1+8.49X2+0.65X3+0.40X4-0.49X12-0.59X22-2.36X32+0.58X42-0.43X1X2-2.18X1X3-0.13X1X4-1.13X2X3+0.14X2X4-0.60X3X4

**Table 3:** Box–Behnken model for uranium(VI) sorption onto IL impregnated-chitosan.

No	Coded variable				Actual variable				Experimental capacity (mg g^-1^)	Predicted capacity (mg g^-1^)
	X_1_	X_2_	X_3_	X_4_	X_1_	X_2_	X_3_	X_4_		
1	-1	-1	0	0	4	20	60	40	9.56	9.26
2	1	-1	0	0	6	20	60	40	9.86	9.52
3	-1	1	0	0	4	60	60	40	27.98	27.11
4	1	1	0	0	6	60	60	40	26.54	25.62
5	0	0	-1	-1	5	40	15	30	14.80	15.52
6	0	0	1	-1	5	40	105	30	19.59	18.02
7	0	0	-1	1	5	40	15	50	17.18	17.52
8	0	0	1	1	5	40	105	50	19.58	17.64
9	-1	0	0	-1	4	40	60	30	17.47	18.82
10	1	0	0	-1	6	40	60	30	18.85	18.46
11	-1	0	0	1	4	40	60	50	18.28	19.88
12	1	0	0	1	6	40	60	50	19.15	19.01
13	0	-1	-1	0	5	20	15	40	6.82	5.74
14	0	1	-1	0	5	60	15	40	23.23	24.98
15	0	-1	1	0	5	20	105	40	9.85	9.31
16	0	1	1	0	5	60	105	40	21.73	24.02
17	-1	0	-1	0	4	40	15	40	15.34	13.58
18	1	0	-1	0	6	40	15	40	17.30	17.32
19	-1	0	1	0	4	40	105	40	19.26	19.24
20	1	0	1	0	6	40	105	40	12.51	14.28
21	0	-1	0	-1	5	20	60	30	9.13	10.19
22	0	1	0	-1	5	60	60	30	28.07	26.89
23	0	-1	0	1	5	20	60	50	9.54	10.73
24	0	1	0	1	5	60	60	50	29.04	27.98
25	0	0	0	0	5	40	60	40	18.78	18.96
26	0	0	0	0	5	40	60	40	18.83	18.96
27	0	0	0	0	5	40	60	40	19.26	18.96

**Figure 3:**
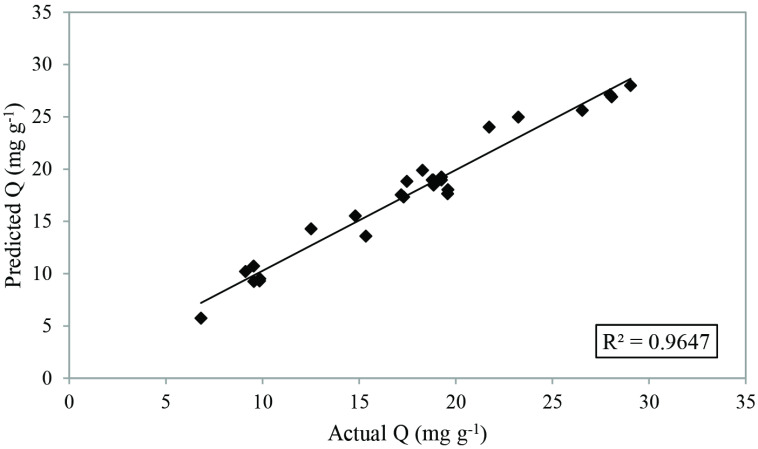
The predicted and actual responses for uranium(VI) sorption.

ANOVA is a statistical method used to check the importance and fitness of the model [32]. Table 4 summarizes ANOVA for uranium(VI) sorption capacity of IL impregnated chitosan. The model F value of 23.43 suggests that the model is important. The capacity values obtained from experimental tests were compared with the predicted ones according to the model. As seen in Figure 3, correlation coefficient (R^2^) value of 0.96 points out the good agreement between experimental and predicted values.

**Table 4:** ANOVA, coefficients, and P-values for uranium(VI) sorption capacity of IL-impregnated chitosan.

ANOVA
	df	Sum of squares	Mean square	F-value	Probability F	R$^{2}$
Regression	14	940.2623	67.16159	23.43	1.49 x 10^-6^	0.96
Residuals	12	34.40155	2.866796			
Total	26	974.6638				
Coefficient	P-value
Intercept	18.96	2.0 x 10^-10^				
X_1_	-0.31	5.4 x 10^-1^				
X_2_	8.49	7.2 x 10^-10^				
X_3_	0.65	2.1 x 10^-1^				
X_4_	0.40	4.2 x 10^-1^				
X_1_X_1_	-0.49	5.1 x 10^-1^				
X_2_X_2_	-0.59	4.4 x 10^-1^				
X_3_X_3_	-2.36	7.4 x 10^-3^				
X_4_X_4_	0.58	4.5 x 10^-1^				
X_1_X_2_	-0.43	6.2 x 10^-1^				
X_1_X_3_	-2.18	2.5 x 10^-2^				
X_1_X_4_	-0.13	8.8 x 10^-1^				
X_2_X_3_	-1.13	2.1 x 10^-1^				
X_2_X_4_	0.14	8.7 x 10^-1^				
X_3_X_4_	-0.60	4.9 x 10^-1^				

Coefficients of independent variables and P-values for the examined parameters (initial pH, contact time, initial concentration, temperature) are also shown in Table 4. The smallest level of importance leading to rejection of the null hypothesis is described as P-value. When P < 0.05, the main effect of each factor and dual effects are regarded as statistically significant [33].

Main effect of initial concentration (P = 7.2 ×10^-10^), quadratic effect of contact time (P = 7.4 ×10^-3^) and dual effect of initial pH and contact time (P = 2.5 ×10^-2^) were found statistically significant (Table 4).

pH is one of the most significant parameters controlling the speciation of uranium(VI) and the surface properties of IL impregnated chitosan. Since speciation depends on pH, adsorbed species are also significantly affected by pH change. Uranium(VI) and UO_2_^2+^ are the most stable oxidation state and chemical form at pH < 5, respectively. Low adsorption capacity at low pH is associated with the competition of H+ ions with uranium(VI) ions on active surface sites [34]. Uranyl ion starts to hydrolyze above pH 3.0 according to Eq. (5) and the amount of UO_2_(OH)^+^, UO_2_(OH)_2_^2+^ and (UO_2_)_3_(OH)^5+^ tend to increase until the UO_2_(OH)_2_ precipitates [35]. Therefore, UO_2_^2+^, UO_2_(OH)^+^, UO_2_(OH)_2_^2+^, and (UO_2_)_3_(OH)^5+^ are the species likely to exist in solution at pH 3.0–5.0.

(5)UO22++nH2O[UO2(H2O)n-m}2-m+mH+

**Figure 4:**
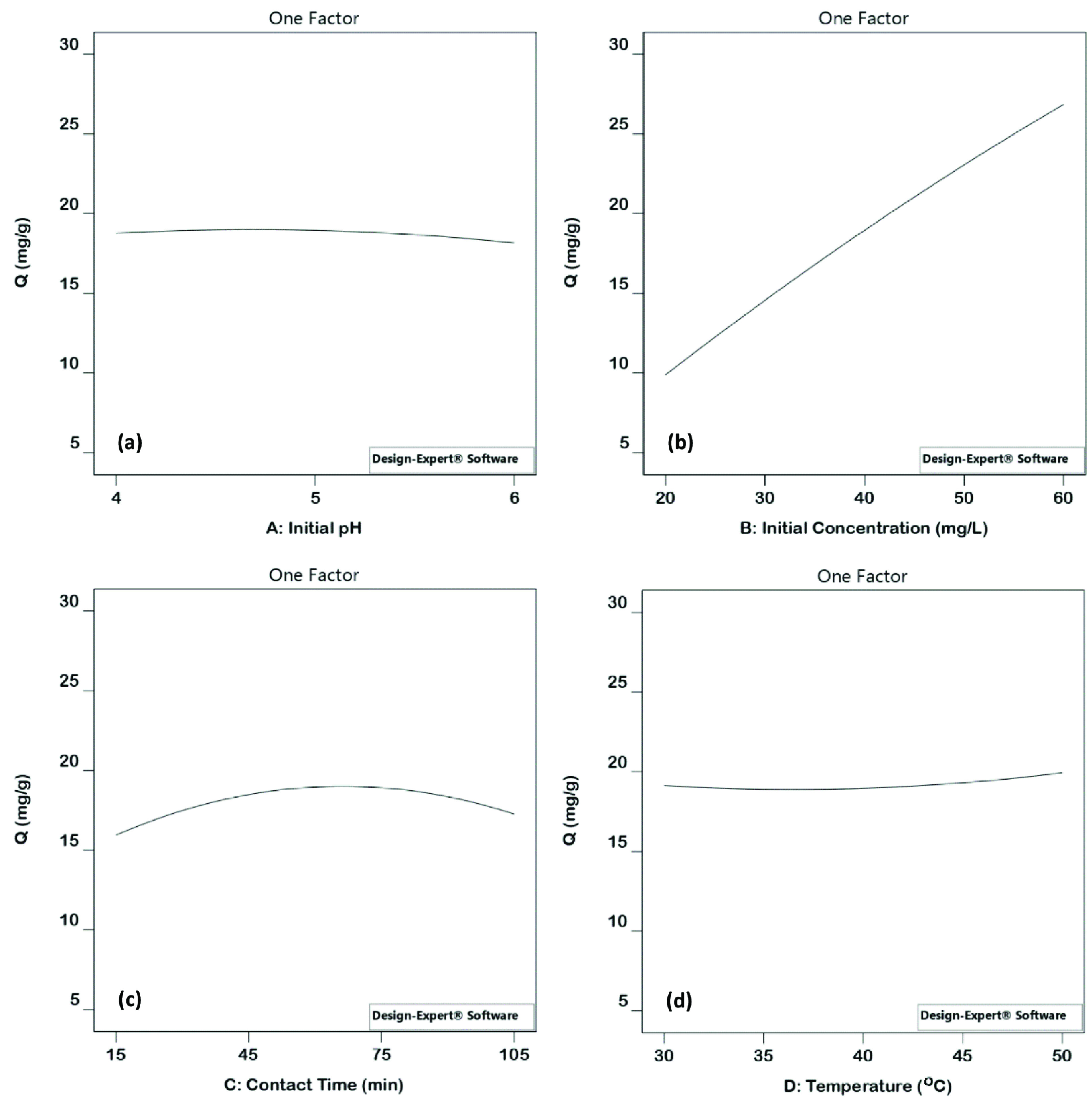
Main effects on uranium(VI) sorption capacity; effect of initial pH (a), effect of initial concentration (b), effect of contact time (c), effect of temperature (d).

Initial pH (X_1_ = – 0.31) has a negative effect on sorption capacity. However, the positive value of the coefficients belonging to initial concentration (X_2_ = 8.49), contact time (X_3_ = 0.65), and temperature (X_4_ = 0.49) indicated that initial concentration, contact time, and temperature have a positive effect on the sorption of uranium(VI). It means that the sorption capacity of uranium(VI) decreases with the increasing initial pH, whereas uranium(VI) capacity increases as initial concentration, contact time, and temperature increase (Figures 4a–4d).

**Figure 5:**
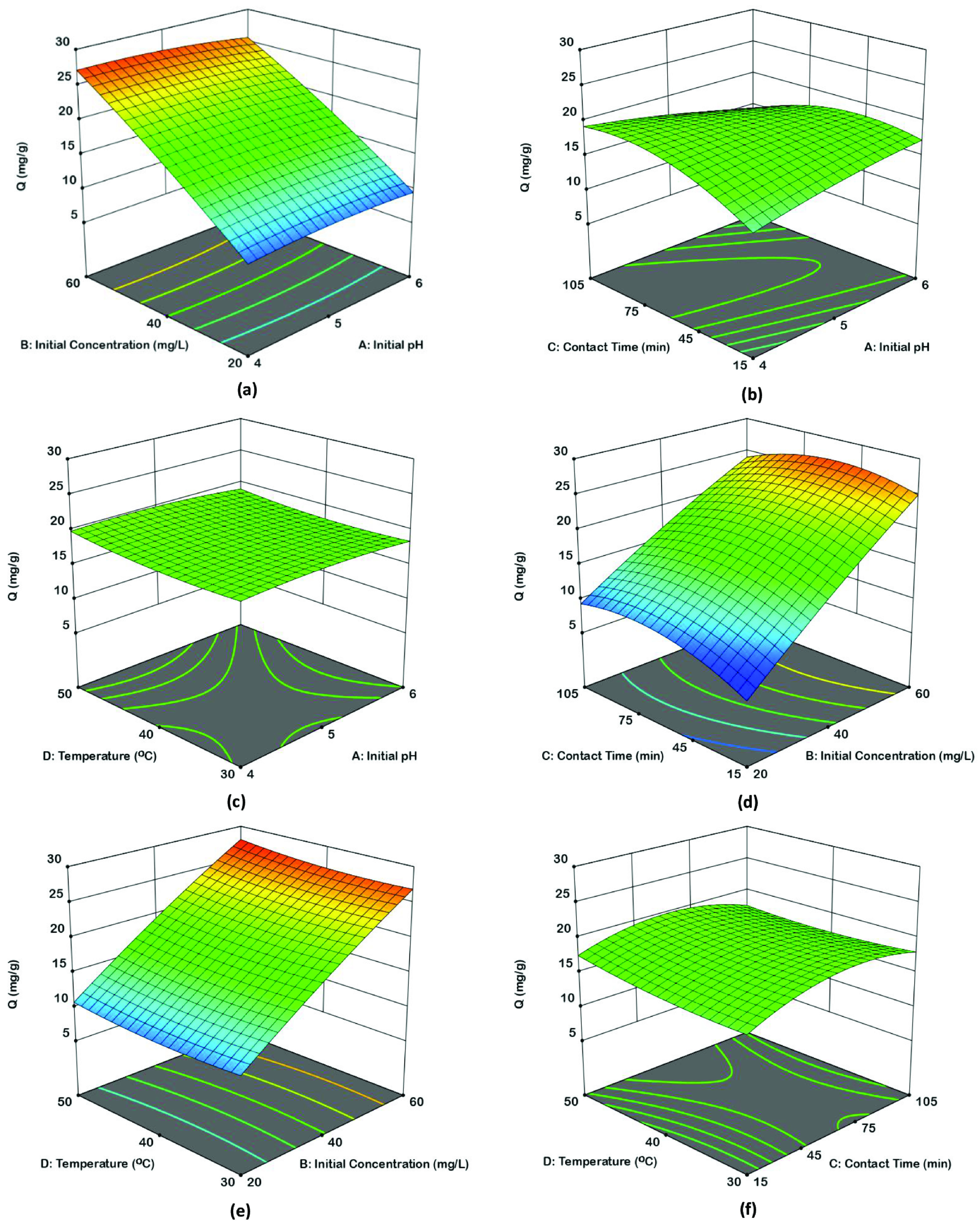
Response surface plots for dual effects; effect of initial pH and initial concentration (a), effect of initial pH and contact time (b), effect of initial pH and temperature (c), effect of initial concentration and contact time (d), effect of initial concentration and temperature (e), effect of contact time and temperature (f).

In order to determine the dual effects of variables on the uranium(VI) sorption capacity, response surface methodology (RSM) has been utilized. Dual effects of independent variables are illustrated with 3D surface plots in Figures 5a–5f. In each 3D surface plot, two variables are altered while other two variables are hold at center levels.

Figure 5a illustrates the dependence of sorption on initial pH (X_1_) and initial concentration (X_2_). Maximum Q was found to be 27.14 mg g^-1^ at initial pH 4.2 and initial concentration of 60 mg L^-1^ by fixing the contact time at 60 min and the temperature at 40 °C. Figure 5b shows the interaction between initial pH (X_1_) and contact time (X_3_). Maximum Q was obtained as 19.62 mg g^-1^ at initial pH 4 and contact time of 87 min by holding the initial concentration and temperature at 40 mg L^-1^ and 40 °C, respectively. Figure 5c presents the dependency of sorption on both initial pH (X_1_) and temperature (X_4_). Maximum Q of 20.04 mg g^-1^ was obtained at initial pH 4.6 and temperature of 50 °C by fixing initial concentration and contact time at center levels. Figure 5d shows the interaction between initial concentration (X_2_) and contact time (X_3_). Maximum Q was achieved as 26.88 mg g^-1^ at 60 mg L^-1^ and 55 min by holding the pH and temperature at center levels. Figure 5e indicates the interaction between initial concentration (X_2_) and temperature (X_4_). Maximum Q was obtained as 27.98 mg g^-1^ at 60 mg L^-1^ and 50 ºC by fixing the pH at 5 and contact time at 60 min. Figure 5f demonstrates the dependence of sorption on contact time (X_3_) and temperature (X_4_). Maximum Q was found to be 19.94 mg g^-1^ at 60 min and 50 °C by fixing initial pH at 5 and initial concentration at 40 mg L^-1^.

### 3.3. Kinetic studies

Kinetic studies were conducted in order to determine the behavior of IL-impregnated chitosan towards uranium ions. Experiments were carried out between 5 and 360 min of contact time. The experimental kinetic data were evaluated using pseudo-first and pseudo-second-order kinetic models. Pseudo-first-order model is expressed as:

(6)dqtdt=k1(qe-qt)

Integrated form of the equation is:

(7)ln(qe-qt)=lnqe-k1t

where k_1_ is the first-order rate constant (min^-1^), *q_t_* and *q_e_* are amount of metal ion sorbed (mg g^-1^) at time *t* and at equilibrium, respectively [36,37]. *k_1_* and *q_e_* can be obtained from the slope and the intercept of the plot. Pseudo-second-order model [38] is based on sorption capacity of solid phase and is given in Eq. (8):

(8)dqtdt=k2(qe-qt)

where *k_2_* is the second-order rate constant (g mg^-1^min^-1^). Integrated linear form of the equation is expressed in Eq. (9):

(9)tqt=1k2qe2+tqe

The experimental data of uranium(VI) sorption can be explained well with pseudo-first-order and pseudo-second order kinetic models. However, pseudo-second-order kinetic model has a slightly higher correlation coefficient value (R^2^ = 0.999). The determined qe values were in accordance with the experimental data.

**Figure 6:**
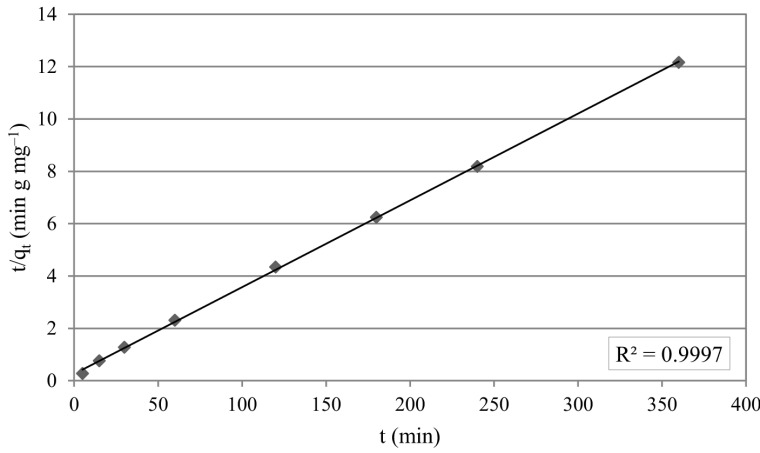
Pseudo-second-order kinetic plot.

The kinetic plot of t/q_t_ versus t for uranium(VI) sorption is presented in Figure 6. q_e_ and k_2_ can be determined from the slope and the intercept of the plot. Pseudo-first-order model parameters q_e_, k_1_, R^2^ and pseudo-second-order model parameters q_e_, k_2_, and R^2^ are displayed in Table 5.

**Table 5:** Kinetic model parameters for uranium(VI) sorption

Kinetic model	k_1_(min^-1^)	q_e_(mg g^-1^)	R^2^
Pseudo-first-order	0.016	11.09	0.991
	k_2_(g mg min^-1^)	q_e_(mg g^-1^)	R^2^
Pseudo-second-order	0.005	30.30	0.999

### 3.4. Sorption isotherms

**Figure 7:**
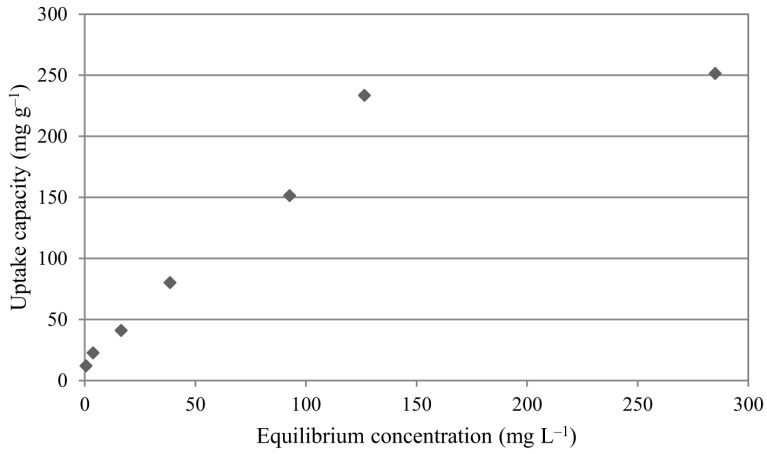
Sorption isotherm of uranium(VI) on IL-impregnated chitosan (initial pH: 4, temperature: 50 °C, contact time: 70 min, sorbent amount: 50 mg).

The effect of initial concentration on uranium(VI) sorption was examined between 25 and 800 mg L^-1^. Sorption isotherm is presented in Figure 7. Uranium(VI) uptake increased from 12.07 to 233.51 mg g^-1^ with a rise in the initial uranium(VI) concentration from 25 to 600 mg L^-1^. After this point, uranium(VI) uptake capacity of the sorbent reached a plateau and remained almost constant. This behavior can be interpreted as the saturation of active sites on the sorbent.

Sorption isotherms determine the relation between quantity of the metal sorbed and the metal concentration in the solution at equilibrium.

According to the Langmuir theory, sorption takes place at specific homogeneous sites on the sorbent. Langmuir model in its linear form [39] is described by Eq. (10):

(10)Ceqe=1qmb+Ceqm

where *q_m_* is the maximum amount of the metal ion per unit weight of sorbent to form a monolayer (mg g^-1^), *C_e_* is the equilibrium concentration of metal ion (mg L^-1^), and *b* is a constant related to the sorption energy (L mg^-1^). *q_m_* and *b* can be determined from the slope and intercept of linear plot between *C_e_/q_e_* and *C_e_* .

*q_m_* , *b* , and correlation coefficient (R^2^) values determined from the isotherm are given in Table 6. *q_m_* value was estimated to be 312.5 mg g^-1^ for uranium(VI).

**Table 6:** Isotherm model data for uranium(VI) sorption onto IL impregnated chitosan

Isotherm model	Parameter	Value
Langmuir	q_m_(mg g^-1^)	312.5
b(L mg^-1^)	0.01
R^2^	0.88
Freundlich	K_f_	8.97
	n	1.62
	R^2^	0.97

Freundlich isotherm points out the multisite adsorption on heterogeneous surfaces. Freundlich adsorption isotherm [40] and its linear form are represented by Eq. (11) and Eq. (12), respectively:

(11)qe=KfCe1n

(12)lnqe=lnKf+1nlnCe

where *q_e_* is the equilibrium sorption capacity (mg g^-1^), *C_e_* is the equilibrium concentration of the metal in solution, *K_f_* and *n* are constants related to sorption capacity and intensity, respectively. The calculated values of *n* and *K_f_* are given in Table 6. Among the adsorption isotherms tested, Freundlich isotherm gave the best fit with a R^2^ value of 0.97.

The comparison of uranium(VI) sorption capacity of poly(ethylene glycol)-based dicationic IL-impregnated chitosan with some other sorbents reported in the literature is presented in Table 7.

**Table 7:** 

Sorbent	Initial pH	Time (min)	Uranium(VI) capacity (mg g^-1^)	Reference
IL-impregnated diatomite	4.2	240	88.00	[41]
Chitosan impregnated with magnetite nanoparticles	5	40	42.00	[42]
Magnetite nanoparticles	7	360	5.00	[43]
DTPA-functionalized magnetic chitosan nano-particles	5	60	157.08	[44]
Cysteine functionalized magnetic chitosan microparticles	3.6	60	99.96	[45]
Ion-imprinted magnetic chitosan resin	5	180	188.02	[46]
Poly(ethylene glycol)-based dicationic IL-impregnated chitosan	4	70	251.52	Present study

Pure chitosan can be used for the sorption of metal ions and as a result of preliminary tests, we have confirmed that it has an affinity for uranium(VI). However, impregnation of IL enhanced the sorption capacity of chitosan for uranium(VI) ions by approximately 25%. From the data in Table 7, it can be seen that the the poly(ethylene glycol)-based dicationic IL-impregnated chitosan has a relatively high absorption capacity of 251.52 mg g^-1^ for uranium(VI) compared to other reported sorbents. Therefore, IL-impregnated chitosan can be used as a high capacity sorbent for the removal of uranium(VI) from acidic aqueous solution.

### 3.5. Effect of competing ions

The effect of competing ions on the selective sorption of uranium(VI) was investigated in mixed ion solutions containing zinc(II) and nickel(II). IL-impregnated chitosan was mixed with the metal mixture solutions of uranium(VI), zinc(II), and nickel(II) at pH 4. In the experiments, concentrations of zinc(II) and nickel(II) were varied between 6 and 60 mg L^-1^ while the concentration of uranium(VI) was kept constant at 60 mg L^-1^. The compositions of test solutions are shown in Table 8.

**Table 8:** 

Solution	Ion	C_O_ (mg L^-1^)	C_e_ (mg L^-1^)	Q (mg g^-1^)	K_d_ (mL g^-1^)	k
A	UO_2_^2+^	60.08	5.27	26.80	5086.44	-
B	UO_2_^2+^	60.4	9.84	24.71	2511.00	-
	Zn^2+^	6.11	5.80	0.15	26.21	95.81
	Ni^2+^	6.00	5.35	0.32	59.29	42.35
C	UO_2_^2+^	60.19	14.89	22.38	1502.90	-
	Zn^2+^	29.80	28.64	0.57	20.07	74.87
	Ni^2+^	29.81	26.05	2.14	71.22	21.10
D	UO_2_^2+^	60.25	17.50	20.85	1191.15	-
	Zn^2+^	60.19	52.7	3.65	69.30	17.19
	Ni^2+^	59.62	49.21	5.08	103.13	11.55

*Initial pH = 4, contact time = 70 min, temperature = 50 °C, V = 25 mL, m = 0.05 g.

The distribution coefficient ( *K_d_* ) is defined as the ratio of metal ion concentration in the solid phase to metal ion concentration in the solution at equilibrium. It is used to estimate the selectivity coefficient ( *k* ) for the sorption of a metal ion in the presence of other competing ions and expressed by Eq. (13) [47]:

(13)Kd=C0-CeCexVm

where *C_0_* and *C_e_* are the initial and equilibrium concentration of ions (mg L^-1^), respectively, *V* is the volume of the solution (mL), and *m* is mass of the sorbent (g).

Selectivity coefficient ( *k* ) for a specific metal ion is determined as follows [48]:

(14)k=Kd(U)Kd(M)

where *K_d_(U)* and *K_d_(M)* are the distribution coefficient of uranium(VI) and competing ion (mL g^-1^), respectively.

The experimental data along with the sorption capacity(Q), distribution coefficient (K_d_) and selectivity coefficient (k) values are presented in Table 8. Although uranium(VI) sorption capacity is slightly decreased with the increase in the concentration of zinc(II) and nickel(II) ions, it is considerably high compared to that of zinc(II) and nickel(II). K_d_ values are in the order of UO_2_^2+^ >Ni^2+^ >Zn^2+^ at all concentration ranges which indicates the affinity of material towards UO_2_^2+^ ion.

According to the k values in Table 8, selectivity order can be given as UO_2_^2+^ >Zn^2+^ >Ni^2+^. These results suggest that IL impregnated chitosan can be used successfully in the treatment of radioactive wastewater polluted with uranium(VI) in the presence of competing ions.

### 3.6. Desorption studies

From an environmental and economic perspective, desorption efficiency and reuse of a sorbent are important parameters for adsorption processes. The desorption of uranium(VI) was investigated by using mineral acids (HNO_3_ and H_2_SO_4_). As a first step, each 50 mg of sorbent was contacted with 60 mg L^-1^ uranium(VI) solution at 50 °C for 70 min. Following solid–liquid separation, the uranium(VI)-loaded sorbent was washed with deionized water and dried at 50 °C.

For desorption step, uranium(VI)-loaded sorbents were contacted with dilute concentrations (0.01–0.5 M) of HNO_3_ and H_2_SO_4_ solutions at 25 °C for 60 min. IL-impregnated chitosan decomposed and dissolved in nitric acid solutions. Therefore, it was concluded that nitric acid is not suitable for desorption in the studied concentration range. On the other hand, IL-impregnated chitosan is insoluble in H_2_SO_4_; thus, H_2_SO_4_ can be used as a desorption agent as reported in literature [49,50]. Desorption efficiency increased with the increase in H2 SO4 concentration. It was determined that 74% and 98% of uranium(VI) ions were desorbed using 0.5 M H_2_SO_4_, in one and two steps, respectively.

## 4. Conclusion

In this study, a novel poly(ethylene glycol)-based dicationic IL-impregnated chitosan was synthesized and characterized by FTIR, SEM-EDX, and TGA analysis. Sorbent was found to be chemically stable in the pH range studied. The effect of initial pH, initial concentration, contact time, and temperature on uranium(VI) sorption capacity was determined using 3 level BBD. Initial concentration, quadratic effect of contact time, dual effect of initial pH and contact time were found statistically significant. High correlation coefficient (R^2^) of 0.96 reveals that actual and predicted sorption capacity values are in a good agreement. The model F value of 23.43 implies that BBD model is significant. 3D surface plots were drawn to analyze the effect of dual interactions on sorption. Maximum uranium(VI) sorption capacity of IL impregnated chitosan was found as 28.48 mg g^-1^ at the following conditions; initial pH, 4; initial concentration, 60 mg L^-1^; contact time, 70 min and temperature, 50 °C. Kinetic data fit the pseudo-second-order kinetic model better. By the evaluation of sorption isotherm, uranium(VI) sorption was found as a Freundlich type isotherm (R^2^ = 0.97). IL-impreganted chitosan has been demonstrated to be a selective sorbent for uranium(VI) ions in the presence of zinc(II) and nickel(II).

It can be said that there is electrostatic and hydrophobic interactions and hydrogen bonding between chitosan and IL. Moreover, impregnated sorbent had an alkaline character before sorption; thus, interaction between sorbent and acidic uranium(VI) solution was electrostatic. Overall results indicate that poly(ethylene glycol) bis(methylimidazolium) di[bis(trifluoromethylsulfonyl)imide] impregnated chitosan can be utilized as a high-capacity and selective sorbent for the removal of uranium(VI) ions in acidic solutions.
